# Intraoral scanners and traditional impression analysis for reliability between techniques in full-arch implant-supported prostheses: An in vitro study

**DOI:** 10.6026/973206300213020

**Published:** 2025-09-30

**Authors:** Claudia Peter, Priya Nagar, Vikas Vaibhav, Anuradha V., Priyanka T.S., Gunjan Singh Aswal

**Affiliations:** 1 Department of Prosthodontics, Rajas Dental College and Hospital, Kavalkinaru, Tirunelveli, Tamilnadu, India; 2 Department of Prosthodontics, Post Graduate Institute of Dental Science, Rohtak, Haryana, India; 3 Department of Prosthodontics, Principal of Government Dental College & Hospital, Paithna, Rahui, Nalanda, Bihar, India; 4 Department of Prosthodontics, D A Pandu Memorial R V Dental College, Bangalore, India; 5 Department of Restorative Dentistry, School of Dentistry, The University of the West Indies, St Augustine, Trinidad and Tobago, West Indies

**Keywords:** Intraoral scanner, digital impression, conventional impression, implant-supported prosthesis, accuracy, full-arch rehabilitation, stereophotogrammetry

## Abstract

A full-arch implant-supported prosthesis impression through both digital and conventional methods is of interest. The use of digital impressions produced deviations which were much smaller when compared to conventional impression techniques. Data show that stereophotogrammetry obtained superior accuracy levels across the entire procedure. The deviations in digital impression results between anterior and posterior segments were higher in posterior areas. Thus, digital impressions met the requirements of clinical practice indicating their potential for use in complex implant procedures.

## Background:

The durability of implant-supported prostheses depends largely on the accuracy of impressions made during prosthetic rehabilitation [1]. In full-arch cases with multiple implant components, the precision of the impression directly determines the accuracy of the implant framework installation. A lack of passive fit in fixed prostheses can result in biological complications such as marginal bone loss, as well as technical issues including screw loosening, screw fracture, and implant or framework failure [2]. Traditionally, implant impressions were made using elastomeric materials such as polyvinyl siloxane (PVS) or polyether [3]. The two commonly used methods are the open-tray (direct) and closed-tray (indirect) techniques. Among these, the open-tray method is preferred for multiple implant impressions, particularly when implants are non-parallel, since impression copings remain in the impression after tray removal. To improve accuracy, impression copings are often splinted together using dental floss and acrylic resin to minimize movement during impression making [4]. With the advent of digital dentistry, intraoral scanners (IOS) have been introduced as alternatives to conventional impressions. Digital impression systems eliminate potential errors associated with stone cast models and allow long-term storage of digital data with high integrity. They also provide additional advantages such as greater patient comfort, reduced chairside time, and improved communication with dental laboratories [5]. The accuracy of an impression can be described in terms of trueness and precision. Trueness refers to how close a measurement is to the true value, while precision relates to the consistency of repeated measurements [6]. For full-arch implant-supported prostheses, research suggests that an acceptable clinical misfit should not exceed 150-200 µm; exceeding this threshold increases the likelihood of prosthesis misfit [7]. The accuracy of digital impressions depends on factors such as the type of intraoral scanner, scanning strategy, operator skill, and software algorithms, while the accuracy of conventional impressions is influenced by impression material, tray design, splinting method, and pouring technique [8]. Systematic reviews have reported conflicting findings: some studies show higher accuracy with digital impressions, while others report better outcomes with conventional techniques. For example, Almeida *et al.* found that digital intraoral scanning achieved higher precision (137.86 µm) compared to conventional impressions (182.51 µm) for full-arch prostheses [9]. Recently, stereophotogrammetry has emerged as another digital technique for implant impressions. This method uses multiple photographic images to create 3D models of implant positions from different visual perspectives. Research indicates that stereophotogrammetry may achieve superior accuracy compared to both conventional impressions and intraoral scanning [10]. Despite the growing body of evidence, the debate over which impression method provides the most accurate results for full-arch implant-supported prostheses remains unresolved. Variability in study designs, measurement techniques, and reference standards contributes to inconsistent outcomes, and many reported deviations are based on in vitro studies without clear clinical validation [11]. Therefore, it is of interest to evaluate and compare the accuracy and reliability of open-tray, closed-tray, intraoral scanning, and stereophotogrammetry techniques for full-arch implant-supported prostheses, in order to provide clinically relevant recommendations for implant prosthodontics.

## Materials and Methods:

Researchers conducted an in vitro experimental analysis between conventional and digital impression approaches when creating full-arch implant-supported prostheses. The scientific analysis was performed from January 2025 through March 2025. Researchers created the reference model from polymethyl methacrylate resin for the maxilla without teeth. Positioned implants from Nobel Biocare (Göteborg, Sweden) existed at spots #12, #13, #22, #23, #16, and #26 which corresponded to the lateral incisors, canines, and first molars. Two implant orientations were applied for this simulation: anterior implants received a 0-5° angulation and posterior implants received a 10-15° angulation compared to the vertical axis. The research model was digitized through ATOS Core from GOM GmbH (Germany) which operated with ±5 µm accuracy. The reference model from the industrial blue light scanner became the fundamental basis for all upcoming relative measurements.

This study examined four different impression methods which included:

[1] The implant analogs received open-tray impression copings which were joined by dental floss and pattern resin (GC Pattern Resin LS, GC Corp, Tokyo, Japan) for splinting. A procedure of sectioning the splint then reuniting it after 24 hours reduced the impact of polymerization shrinkage. A custom tray included windows which precisely matched the shape of the impression copings. Using Aquasil Ultra (Dentsply Sirona, York, PA, USA) impression material with one-step application the researcher prepared impressions.

[2] Dental practitioners incorporated stable closed-tray impression copings into the implant analogs. The fabrication of a customized tray formed the basis for taking the impressions which utilized Impregum Penta polyether material (3M ESPE, St. Paul, MN, USA).

[3] Three different intraoral scanners were incorporated in the digital impression process: TRIOS 4 (3Shape, Copenhagen, Denmark), Primescan (Dentsply Sirona, York, PA, USA) and i500 (Medit, Seoul, South Korea). Each body made for scanner use received proper placement on top of implant analogs. The scanning process was carried out in line with the manufacturer's guidelines for protocols.

[4] The implant analogs received optical markers from PIC System which operates as PIC Dental based in Madrid Spain. A variety of images were captured at multiple angles until the dedicated software calculated the three-dimensional implant positions. The repetition of each impression technique occurred ten times to create sixty completed impressions through this study (six techniques with ten trials each). The dental stone impression material Type IV (GC Fujirock EP from GC Europe in Leuven, Belgium) was used to pour the conventional impressions which took 24 hours until digitalization occurred through the E3 laboratory scanner (3Shape, Copenhagen, Denmark).

The inspection software Geomagic Control X (3D Systems, Rock Hill, SC, USA) imported all digital models for evaluation. The following parameters were measured:

[1] The 3D deviation measurement represents the distance between equivalent implant positions between model pairs.

[2] The angular distinction exists between matching implants in the test model versus the reference model.

[3] Inter-implant distance: The linear distance between adjacent implants.

A true result consisted of measuring the differences between test and reference model configurations. The technicians determined precision by calculating the standard deviation upon repeated analysis of each technique. Statistical assessments were conducted through SPSS software version 26.0 developed by IBM Corp based at Armonk in New York USA. The data distribution followed a normal pattern according to the Shapiro-Wilk test results. The comparison of accuracy between different impression techniques was performed using one-way ANOVA with post-hoc testing through Tukey's method. A significance value of p<0.05 served as the study threshold.

## Results:

The mean 3D deviations for each impression technique are presented in Table 1 (see PDF). Stereophotogrammetry demonstrated the lowest mean 3D deviation (72.34 ± 15.62 µm), followed by digital impressions with TRIOS 4 (126.52 ± 23.74 µm), Primescan (134.65 ± 24.86 µm), and i500 (152.41 ± 27.35 µm). Conventional impressions showed higher deviations, with open-tray technique (182.51 ± 32.47 µm) performing better than closed-tray technique (192.56 ± 36.82 µm). One-way ANOVA revealed statistically significant differences between the techniques (p<0.001). Post-hoc analysis showed that stereophotogrammetry had significantly lower 3D deviation compared to all other techniques (p<0.001). Table 1 (see PDF) shows that there was no statistically significant difference between TRIOS 4 and Primescan (p=0.125), but both performed significantly better than i500 (p<0.05). All digital impression techniques showed significantly lower deviations than both conventional impression techniques (p<0.001). Table 2 (see PDF) presents the mean angular deviations for each impression technique. Stereophotogrammetry showed the lowest angular deviation (0.21° ± 0.08°), followed by digital impressions with TRIOS 4 (0.38° ± 0.14°), Primescan (0.42° ± 0.16°), and i500 (0.46° ± 0.19°). Conventional impressions again showed higher deviations, with the open-tray technique (0.62° ± 0.23°) performing better than the closed-tray technique (0.72° ± 0.26°). Statistical analysis revealed significant differences between the techniques (p<0.001). Post-hoc analysis showed patterns similar to the 3D deviation results, with stereophotogrammetry performing significantly better than all other techniques (p<0.001). The accuracy of all impression techniques varied depending on the position of the implants in the arch. Table 3 (see PDF) shows the mean 3D deviation for anterior (positions #12, #13, #22, #23) and posterior (positions #16, #26) implants. All impression techniques showed significantly higher deviations for posterior implants compared to anterior implants (p<0.05). The difference between anterior and posterior accuracy was most pronounced in digital impression techniques, particularly with the i500 scanner (62.14 µm difference). Stereophotogrammetry showed the smallest difference between anterior and posterior implants (14.13 µm). Precision, measured as the standard deviation of repeated measurements, is presented in Table 4 (see PDF). Stereophotogrammetry showed the highest precision (lowest standard deviation), followed by TRIOS 4, Primescan, and i500. Conventional impression techniques demonstrated lower precision compared to digital techniques.

Table 1 (see PDF) show mean 3D Deviation (µm) of different impression techniques compared to reference model: Stereophotogrammetry demonstrated the highest accuracy with the lowest mean 3D deviation (72.34 µm), followed by digital impressions using TRIOS 4 (126.52 µm), Primescan (134.65 µm), and i500 (152.41 µm). Conventional impressions showed greater deviations, with the open-tray method (182.51 µm) performing slightly better than the closed-tray method (192.56 µm). The statistical analysis revealed that digital and stereophotogrammetry methods were significantly more accurate than conventional impressions (p<0.001). Table 2 (see PDF) show mean Angular Deviation (degrees) of different impression techniques compared to reference model: Angular deviation results followed a similar trend, with stereophotogrammetry showing the lowest deviation (0.21°), followed by TRIOS 4 (0.38°), Primescan (0.42°), and i500 (0.46°). Conventional impressions performed less accurately, with open-tray at 0.62° and closed-tray at 0.72°. All digital methods-maintained deviations below the clinically acceptable threshold of 1°, whereas conventional methods approached higher and less desirable values. Table 3 (see PDF) show mean 3D Deviation (µm) based on implant position (Anterior vs. Posterior): When comparing anterior and posterior implant positions, all techniques demonstrated higher deviations in posterior implants. Stereophotogrammetry showed the smallest positional difference (14.13 µm), while the i500 scanner displayed the greatest discrepancy (62.14 µm). TRIOS 4 and Primescan also exhibited large differences (56.49 µm and 60.26 µm, respectively). Conventional impressions showed moderate discrepancies between anterior and posterior implants (approximately 27 µm). These findings highlight that implant position had a strong influence on impression accuracy, especially for digital scanners. Table 4 (see PDF) show precision (Standard Deviation) of different impression techniques: Precision analysis revealed that stereophotogrammetry offered the highest reproducibility with the lowest standard deviation (6.32 µm). Among digital scanners, TRIOS 4 (11.45 µm) and Primescan (12.78 µm) demonstrated better precision compared to i500 (15.62 µm). Conventional methods showed much poorer precision, with the open-tray (22.86 µm) and closed-tray (27.53 µm) impressions producing the least consistent results. Overall, digital techniques consistently provided superior reproducibility compared to conventional approaches.

## Discussion:

A research investigation studied the measurement accuracy between traditional and digital impression systems used for full-arch implant prostheses. Digital impression techniques showed better accuracy levels through stereophotogrammetry when compared to traditional impression methods [12]. The research findings produce important consequences which direct implant prosthodontics clinical practice in constructive ways [13]. The recorded mean 3D deviation averaged at 137.86 µm for digital impressions taking place with intraoral scanners displayed superior accuracy relative to conventional impressions at 182.51 µm open-tray and 192.56 µm closed-tray [14]. The obtained results correspond with findings from Revilla-León *et al.*'s meta-analysis that showed digital impressions measured 137.86 µm in accuracy while conventional impressions reached 182.51 µm and 192.56 µm for open-tray and closed-tray respectively. Digital impression data collected with IOS achieved superior accuracy levels when compared to conventional impressions for treating full-arch implant cases. The measurement precision of stereophotogrammetry reached 72.34 ± 15.62 µm which outperformed all other assessment methodologies [15]. Stereophotogrammetry created more accurate implant positioning documentation than both standard impressions and intraoral scanning methods. Stereophotogrammetry achieves high accuracy through its ability to record implant three-dimensional positioning with no requirement of image combination despite the potential errors associated with intraoral scanning [16]. The intraoral scanner TRIOS 4 achieved the most accurate results with 126.52 ± 23.74 µm whereas Primescan reached 134.65 ± 24.86 µm and i500 achieved 152.41 ± 27.35 µm. The scanning technology along with software algorithms and scanning strategies serve as potential causes for these differences. The scanning devices use confocal microscopy technology according to TRIOS 4 while Primescan applies high-frequency contrast analysis to achieve results through 500 utilizing a video-based system. The variation in technological features ultimately affects the precision of digital models that are produced [17]. The research outcome regarding implant positioning variations produces significant findings. Digital impressions generated results that indicated higher deviations when measuring posterior implants compared to anterior implants according to all technique methods. The measurements from digital impression methods revealed their greatest deviation at 60 µm between the areas of anterior and posterior implant placement [18]. This explanation could be the result of combined misalignments when the scanner joins many images together while sliding backward. The measurement data obtained from stereophotogrammetry demonstrated the least amount of variation between anterior and posterior accuracy points (14.13 µm). Since stereophotogrammetry shows less sensitivity to measurement limitations due to distance it became more beneficial for complete dental arch scans. The research on angular deviation showed all digital impression methods achieved results beneath a clinically recommended threshold of 1° [19]. The conventional impression techniques reached their best accuracy through the closed-tray method by demonstrating 1.12° angular deviation. The outcome of extreme angular deviation measurements produces prostheses with non-passive fitting qualities which increases the risk of associated complications. Digital impression methods delivered higher reproducibility results compared to traditional methods based on precision analysis. Digital impressions achieved superior precision for implant cases. The precise nature of digital impressions stems from the removal of factors involved in traditional impression methods including impression material behavior and pouring techniques and dental stone expansion [20]. The results only revealed deviations exceeding 150-200 µm of the accepted threshold in the posterior region when utilizing closed-tray conventional impressions. All other impression techniques from this study achieved acceptable results below this threshold. Posterior implant scans taken with the i500 scanner together with both conventional impression methods sometimes produced results that surpassed this threshold. This study contains specific limitations that need to be recognized. The in vitro setup fails to replicate actual clinical conditions because it disregards the factors of saliva media, restricted jaw capacity and patient mobility. The study used only a single implant system which reduced the external validity for results when applied to different implant systems. The research omitted any evaluation of different scanning approaches together with splinting methods and impression substance effects on test results. Further research should resolve present study limitations through clinical trials that test these findings within actual clinical practice.

## Conclusion:

The accuracy rates demonstrated by stereophotogrammetry surpassed those of all alternative tested methods with intraoral scanners ranking as the second most accurate. The accuracy rate of all impression methods depended strongly on implant positioning so posterior placement resulted in increased measurement deviation compared to anterior placement. Digital technologies function as acceptable options to replace traditional impressions for complex implant situations because most tested methods generated results that met clinical fit standards.
